# Utilizing Esophageal Motility Tests in Diagnosing and Evaluating Gastroesophageal Reflux Disease

**DOI:** 10.3390/diagnostics14141467

**Published:** 2024-07-09

**Authors:** Wangliu Yang, Yurong Huang, Lei He, Dongmei Chen, Sheng Wu, Yan Tian, Juan Zheng, Jie Yang, Gengqing Song

**Affiliations:** 1 Department of Gastroenterology, The Affiliated Hospital of Guizhou Medical University, Guiyang 550004, China; yangwl108@163.com (W.Y.); m18185829991@163.com (Y.H.); hl1472583692024@163.com (L.H.); 13765449900@163.com (J.Z.); 2 Department of Gastroenterology, Liupanshui Municipal People’s Hospital, Liupanshui 553000, China; cdm2117@163.com (D.C.); www5454sss@163.com (S.W.); 13639190099@163.com (Y.T.); 3 Department of Gastroenterology and Hepatology, Metrohealth Medical Center, Case Western Reserve University, Cleveland, OH 44106, USA

**Keywords:** gastroesophageal reflux disease, high-resolution esophageal manometry, esophageal motility, endoscopic functional lumen imaging probe, esophagogastric junction contractile integral

## Abstract

Gastroesophageal reflux disease (GERD), a prevalent clinical condition, is often attributed to aberrant esophageal motility, leading to gastric content reflux and associated symptoms or complications. The rising incidence of GERD presents an escalating healthcare challenge. Endoscopic and esophageal reflux monitoring can provide a basis for the diagnosis of patients with gastroesophageal reflux disease, but when the diagnostic basis is at an inconclusive value, some additional supportive evidence will be needed. Advanced technology is the key to improving patient diagnosis, accurate assessment, and the development of effective treatment strategies. High-resolution esophageal manometry (HREM) and endoscopic functional lumen imaging probe (EndoFLIP) represent the forefront of esophageal motility assessment. HREM, an evolution of traditional esophageal manometry, is considered the benchmark for identifying esophageal motility disorders. Its widespread application in esophageal dynamics research highlights its diagnostic significance. Concurrently, EndoFLIP’s emerging clinical relevance is evident in diagnosing and guiding the treatment of coexisting esophageal motility issues. This review integrates contemporary research to delineate the contributions of HREM, EndoFLIP, and novel technologies in GERD. It examines their efficacy in facilitating an accurate diagnosis, differentiating similar gastrointestinal disorders, quantifying the extent of reflux, assessing the severity of the disease, forecasting patient responsiveness to proton pump inhibitor therapy, and guiding decisions for surgical interventions. The overarching aim is to deepen the understanding of GERD’s underlying mechanisms and advance the formulation of holistic, efficacious treatment approaches.

## 1. Introduction

Gastroesophageal reflux disease (GERD) is characterized by the retrograde flow of gastric contents into the esophagus, leading to clinical manifestations and potential complications [1]. The hallmark symptoms of GERD, such as heartburn and regurgitation, signify its impact on patient well-being. GERD encompasses a spectrum of phenotypes based on esophageal mucosal changes: reflux esophagitis (RE), characterized by visible erosion or ulceration of the esophageal lining; non-erosive reflux disease (NERD), where such mucosal damage is not observed; and Barrett’s esophagus, a condition marked by metaplastic changes in the esophageal lining, reflecting a response to chronic reflux. Recognizing these distinct phenotypes is vital for developing personalized treatment approaches and deepening the understanding of GERD’s diverse pathophysiological mechanisms.

**Figure 1:**
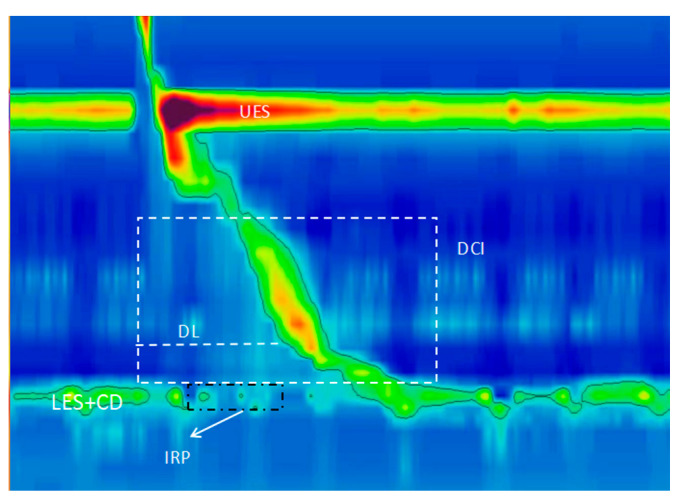
Depiction of normal wet swallows (5 mL) and key esophageal parameters. This figure illustrates a standard swallowing sequence. Following voluntary swallowing, oral and pharyngeal muscles contract, leading to the coordinated opening of the upper esophageal sphincter (UES) and the lower esophageal sphincter (LES). Subsequently, the esophageal body exhibits a sequential peristaltic motion from proximal to distal regions, facilitating the transport of food towards the stomach. Key parameters include UES (upper esophageal sphincter), LES (lower esophageal sphincter), CD (crural diaphragm), DCI (distal contraction integral), IRP (integrated relaxation pressure), and DL (distal latency).

Reflux monitoring, pH-impedance monitoring, or wireless pH monitoring provides an objective assessment of reflux parameters, which is crucial for patients with proton pump inhibitor (PPI) refractory symptoms or when endoscopy is also normal and allows for an assessment of the degree, height, and type (acidic or weakly acidic) of gastroesophageal reflux and the correlation between symptoms and reflux events. Accurate placement of the pH probe is critical to avoid misplacement into the stomach due to esophageal shortening during swallowing. It is recommended to position the probe 5 cm above the lower esophageal sphincter (LES) to ensure reliability [2]. High-resolution esophageal manometry (HREM) offers a quantitative and visual analysis of esophageal dynamic parameters (Figure 1), which is essential for the precise positioning of reflux monitoring devices [3,4,5]. The importance of HREM in understanding GERD’s pathophysiology and diagnosing the condition is increasingly recognized. HREM is particularly valuable (Table 1) for assessing esophageal function and providing guidance in the diagnosis, differential diagnosis, and management of refractory GERD (rGERD). It is especially useful for patients who do not respond adequately to PPI therapy or those whose symptom improvement does not exceed 50% [6].

Endoscopic functional lumen imaging probe (EndoFLIP) is a diagnostic tool engineered to assess the distensibility of the esophagogastric junction (EGJ). Its primary application lies in quantifying the functional parameters of the esophagus in various esophageal disorders. Despite the current guidelines not endorsing the routine application of EndoFLIP for GERD diagnosis [7], its emerging significance in informing and optimizing anti-reflux surgical strategies is garnering notable interest in the clinical community.

## 2. Understanding Pathophysiologies of GERD Based on HREM

### 2.1. Impairment of the Anti-Reflux Barrier at the Esophagogastric Junction (EGJ)

The anti-reflux barrier is a high-pressure zone between the stomach and the esophagus mainly composed of the LES, the CD, and the gastro-esophageal flap valve. The pathophysiology of GERD is multifactorial, and one of the pathophysiological defects in GERD is the compromised efficacy of the EGJ as a barrier to reflux [8]. This dysfunction leads to increased acidic exposure in the distal esophagus, resulting in mucosal damage, such as erosion and ulceration, collectively termed reflux esophagitis (RE). Current HREM methodologies, however, lack a singular comprehensive metric to evaluate EGJ function. Several research groups proposed that esophagogastric junction contractile integral (EGJ-CI) and the LES-CD separation could be used to evaluate the anti-reflux barrier at EGJ, one assessing the anatomical structure of the EGJ and the other its contractile function after research [9,10,11,12,13].

#### 2.1.1. Abnormal Morphology of the EGJ

**Figure 2:**
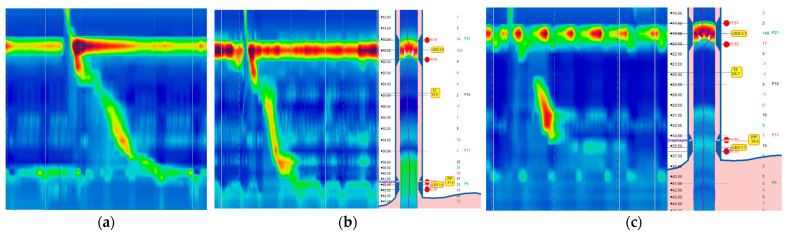
Classification of EGJ Morphology via HREM. This figure categorizes the EGJ into three distinct subtypes based on the spatial relationship between the LES and CD. (**a**) Type I EGJ demonstrates an almost overlapping position of the LES and CD, indicating minimal to no separation. (**b**) Type II EGJ is characterized by a discernible separation between the LES and CD, with RIP between the LES and CD. (**c**) Type III EGJ exhibits a separation of LES and CD, and RIP is proximal to the LES.

EGJ is a multifaceted sphincter composed of the lower esophageal sphincter (LES) and the crural diaphragm (CD). The morphological configuration of the EGJ is defined by the spatial relationship between the LES and CD. HREM serves as a dependable technique for evaluating this morphology, effectively delineating the LES-CD separation. Chicago Classification v4.0 [14,15] mentioned that HREM categorizes the morphology of the EGJ into three distinct subtypes based on the LES and CD’s relative positioning (Figure 2).

In type I, no hiatal hernia (HH), LES, and CD positions largely coincide.

In type II, HH indeterminant, LES, and CD are separated, and respiratory inversion point (RIP) between the LES and CD.

In type III, an HH is present; the separation between LES-CD and RIP is proximal to the LES.

HREM demonstrates enhanced specificity for identifying HH compared to endoscopic evaluations [16,17,18].

The diminished synergistic anti-reflux barrier effect between the LES and the CD at HH is a significant factor in the development of GERD. An HH separates the LES from the CD and is prone to GERD by weakening the anti-reflux barrier [18]. Fuchs et al. conducted an extensive study with 728 GERD patients and identified that HH was the predominant anatomical alteration linked to pathological reflux in 95.4% of cases, followed by LES insufficiency in 88% [19]. Moreover, a 2015 study observed an elevated occurrence of HH in GERD patients (30%) in contrast to healthy individuals (7%) [20]. Tolone et al. [21] noted a correlation between increasing grades of EGJ classification and the incidence of reflux esophagitis; specifically, patients with Type III EGJ exhibited more frequent reflux episodes, prolonged acid exposure, and stronger symptom association compared to those with Types I and II EGJ. Furthermore, Wallner et al. [22] demonstrated that an increased LES-CD separation distance, particularly in sliding HH beyond 2 cm, positively correlates with the severity of reflux-related symptoms. These findings collectively affirm the integral role of HH in the pathogenesis of GERD.

#### 2.1.2. Contractile Capacity of the EGJ

At present, key metrics for assessing EGJ functionality include the integrated relaxation pressure (IRP) and LES pressure integral, which encompasses the mean basal and end-expiratory LES pressures. Hoshino et al. [23] introduced the LES pressure integral as an indicator of distal esophageal pressure, though its accuracy can be influenced by respiratory rate variations. The IRP, measured as the mean pressure during the lowest 4 s period of EGJ relaxation relative to gastric pressure post-LES relaxation, primarily reflects the EGJ’s relaxation ability during swallowing rather than its anti-reflux barrier function. Chicago Classification version 4.0 [14] recommends evaluating the EGJ complex during quiet breathing at baseline in phases devoid of swallowing or recording artifacts.

The EGJ-CI, an HREM-derived metric, evaluates the barrier function of the EGJ, factoring in both its contractile strength and length, independent of respiratory rate influences. This is calculated by adjusting the distal contraction integral (DCI) box to encompass exactly three respiratory cycles of the LES and CD, followed by dividing the resultant DCI value by the duration of these cycles, yielding the EGJ-CI in mmHg·cm [14]. The DCI quantifies the contraction intensity of the distal esophagus from the movement area to the upper boundary of the LES, measured over the duration of esophageal contraction wave pressure transmission, expressed in mmHg·s·cm. The emergence of EGJ-CI as a parameter holds substantial significance in the evaluation of gastroesophageal reflux disease.

- It aids in the diagnosis of GERD; Hyoju et al. [11] identified that an EGJ-CI threshold of 29.5 mmHg cm was indicative of GERD, demonstrating a sensitivity of 77.8% and a specificity of 81.7%. Additionally, they found that an EGJ-CI value of 47 mmHg cm was optimal for GERD diagnosis, achieving a sensitivity of 54% and a specificity of 85% [24];
- It distinguishes between differential diagnoses. The EGJ-CI has also been instrumental in differentiating between patients with functional heartburn (FH) and those with rGERD. It was observed that the rGERD group had a significantly lower EGJ-CI (averaging 25.8 mmHg·cm) in comparison to the FH group, which averaged 39.2 mmHg·cm [25]. This distinction underscores the utility of EGJ-CI for differentiating between these two conditions, aiding in accurate diagnosis and subsequent treatment planning;
- It aids in the prediction of acid reflux. Emerging evidence indicates that patients with abnormal acid exposure time (AET) exhibit lower EGJ-CI values compared to those with normal AET [26,27]. A specific EGJ-CI threshold of 39.3 mmHg·cm has been identified as predictive of abnormal acid exposure, demonstrating a sensitivity of 0.65 and specificity of 0.57 [28]. Current research links diminished EGJ-CI values to increased severity of reflux, suggesting that lower EGJ-CI readings correlate with more pronounced EGJ dysfunction [15].

It is noteworthy that when the separation between the LES and CD is equal to or greater than 3 cm, Gyawali et al. [29] advise excluding the CD component in EGJ-CI calculations. Rogers et al. propose that utilizing a range of values rather than a fixed value for EGJ-CI may provide a more nuanced understanding of the EGJ barrier [30].

EGJ-CI possesses distinctive benefits in the prediction of acid reflux, the measurement of anti-reflux barrier function, aids in the diagnosis of GERD, and for differential diagnosis. However, the establishment of EGJ-CI as the threshold for EGJ dysfunction is still evolving, and variability in its calculation methodology limits its clinical application (Table 2). Further research on EGJ-CI, employing consistent pressure measurement equipment, standardized operational and calculation methods, and encompassing large-scale, multi-center studies, are essential to optimize the clinical efficacy of EGJ-CI in GERD diagnosis.

### 2.2. Transient Lower Esophageal Sphincter Relaxation (TLESR)

**Figure 3:**
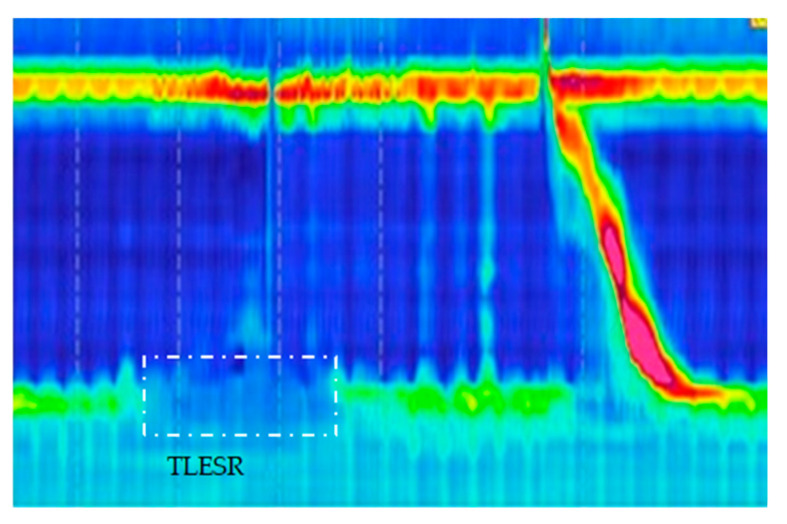
Characterization of TLESR. Depicts TLESR as a phenomenon where there is an absence of swallowing activity in the pharynx 4 s prior to and 2 s following LES relaxation, with these relaxation episodes exceeding 10 s in duration.

TLESR is widely acknowledged as a primary factor in the pathogenesis of gastroesophageal reflux, which is corroborated by multiple studies [34]. It is defined by a relaxation of the LES that occurs independently of swallowing, persists for over 10 s, and frequently involves the suppression of CD activity [35] (Figure 3). Notably, TLESR is observed in both individuals with intact LES function and those diagnosed with GERD. Research by Kim et al. indicates that the occurrence and average duration of TLESR episodes do not significantly differ between GERD patients and healthy controls [36]. Nevertheless, in GERD patients, TLESR episodes are more likely to be associated with gastric acid reflux events [37,38].

In contrast, Schneider et al. [39] reported a doubled frequency of TLESR in GERD patients compared to healthy subjects. Although there remains some debate regarding the increased frequency of TLESR in GERD patients, its physiological mechanism has been leveraged for developing pharmacological treatments like Baclofen. This γ-aminobutyric acid (GABA) receptor agonist, commonly used for CNS relaxation, works by inhibiting vagally induced LES relaxation, thus alleviating GERD symptoms [40].

### 2.3. Motor Disorders in the Esophageal Body

**Figure 4:**
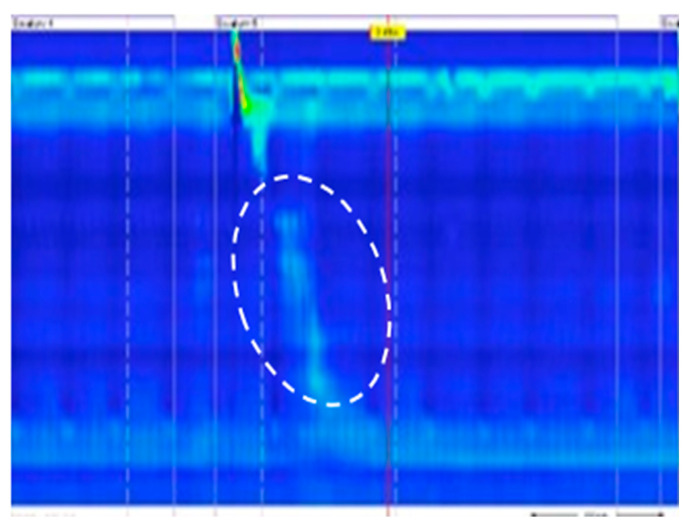
Absence contractility where the DCI (inside the white dotted line circle) < 100 mmHg·s·cm.

A primary functional abnormality in GERD is esophageal hypomotility, which frequently contributes to or exacerbates the condition. In cases where the EGJ outflow is unobstructed, the efficiency of esophageal peristalsis becomes a critical determinant of esophageal clearance. Dysfunctions in esophageal motility lead to compromised clearance, resulting in prolonged exposure of the esophageal mucosa to gastric reflux, which can precipitate GERD. Among GERD patients, ineffective esophageal motility (IEM) is characterized by over 70% of peristaltic contractions being ineffective (Figure 4), or at least 50% of contractions failing, which includes weak contraction (100 mmHg·s·cm ≤ DCI < 450 mmHg·s·cm), complete contraction failure (DCI < 100 mmHg·s·cm), or fragmented contractions [41].

Recent studies suggest that IEM occurrence in GERD patients ranges from 8.8% to 59% [19,26,42,43,44]. GERD patients with IEM, compared to those with normal HREM findings, tend to experience more frequent abnormal reflux episodes (22.7% vs. 9.0%) and demonstrate increased AET and total reflux events [45]. And, LA grade B–D esophagitis and short-segment Barrett’s esophagus are more common in patients with severe IEM and lack of contractility.

## 3. Auxiliary Diagnosis of GERD Based on HREM

The updated Lyon Consensus 2.0 [46] delineates that LA grades B, C, and D esophagitis, biopsy-proven Barrett’s esophagus, and peptic stricture are conclusive for a diagnosis of GERD based on endoscopy. However, most symptomatic patients exhibit normal esophageal mucosa under endoscopy. Esophageal reflux monitoring, including both catheter-based and wireless pH monitoring, remains the gold standard for diagnosing GERD. Findings from pH-impedance monitoring or wireless pH monitoring can confirm the presence or absence of GERD, particularly when the endoscopic results are normal. The updated Lyon Consensus 2.0 proposes that a total acid exposure time (AET) greater than 6% off proton pump inhibitor (PPI) therapy on ambulatory pH-impedance monitoring, or an AET greater than 6% on at least two days of wireless pH monitoring, is conclusive for a GERD diagnosis. However, for AET values ranging from 4% to 6% or total reflux episodes between 40 and 80 per day, and for LA grade A esophagitis, additional supportive evidence is required to make a definitive diagnosis. The Lyon Consensus 2.0 [46] further suggests that supplementary diagnostic evidence can be derived from esophageal manometry data. This includes reduced EGJ pressure [26], the presence of HH [27,47], or IEM/absent contractility [48]. These manometric findings can substantially reinforce the auxiliary diagnosis of GERD, providing a more comprehensive and accurate assessment.

## 4. Differential Diagnosis of Esophageal Motility Disorders Related to GERD Based on HREM

GERD symptoms may originate from both GERD-specific and non-GERD etiologies. HREM is instrumental for identifying esophageal motility disorders that mimic GERD symptoms but are indicative of distinct non-reflux esophageal pathologies, such as achalasia of cardia (AC), esophagogastric junction outlet obstruction (EGJOO), esophageal hypermotility, and distal esophageal spasm (DES) [49]. Advanced diagnostic techniques in HREM, including the rapid drink challenge (RDC), solid test meal (STM), and multiple rapid swallows (MRS), offer critical diagnostic insights into various esophageal motility disorders, enabling the assessment of esophageal contractile reserve, EGJ outflow-tract disorders, and IEM [50,51]. Furthermore, HREM is effective in elucidating esophageal symptoms that are unresponsive to PPI therapy and those not identifiable via endoscopy or barium studies, such as upper gastric belching and Rumination syndrome [52]. Additionally, severe dyskinesia was identified in eight patients (9.75%), including six with AC and two with DES. Of 74 patients undergoing 24-h pH testing, 18 showed no significant acid reflux (DeMeester score < 14.7), with diagnoses including allergic esophagus (*n* = 12) and functional heartburn (FH) (*n* = 6) [53]. This highlights the critical role of esophageal manometry in the differential diagnosis of refractory reflux symptoms.

## 5. Guiding Anti-Reflux Surgical Treatment of GERD Based on HREM

GERD management encompasses diverse approaches, including lifestyle modifications pharmacotherapy, endoscopic techniques, and surgical interventions [54,55]. Surgical intervention is often indicated for patients partially responsive or unresponsive to medication, those experiencing reduced quality of life due to long-term medication use, complications like Barrett’s esophagus, or those with extra-esophageal symptoms linked to GERD [56].

HREM has emerged as a crucial preoperative evaluation tool for endoscopic and surgical treatment of reflux disease [55,57]. Its dual objectives include (1) differentiating GERD-related diseases (e.g., severe esophageal motility disorder, Rumination syndrome, epigastric/gastric belching) from other symptoms and (2) identifying major dynamic disorders to ascertain the suitability of fundoplication, particularly in conditions like AC, DES, and poor esophageal peristaltic reserve, which may increase the risk of postoperative dysphagia [58].

In patients with rGERD undergoing anti-reflux surgery, incorporating MRS in preoperative HREM assessments helps evaluate esophageal contraction reserve. This facilitates the customization of fundoplication procedures (complete or partial wrapping) to minimize postoperative dysphagia risk [50,59]. Routine preoperative pressure measurements serve as a screening tool for identifying GERD patients who might benefit from surgical interventions. In cases with low EGJ-CI, abnormal esophageal exposure to acid, erosive esophagitis, typical GERD symptoms, the response to anti-reflux medications, HH, and absence of esophageal outflow obstruction, anti-reflux therapy has shown enhanced effectiveness [26,31,60]. Furthermore, HREM is integral not only for preoperative assessments but also for postoperative dysphagia evaluations. Identifying dual high-pressure zones (DHPZ) on post-fundoplication HREM serves as a key predictive marker for GERD recurrence or persistence [61,62]. Additionally, an increase in preoperative IRP is a reliable predictor of postoperative dysphagia [63].

In a study by Wang et al. [64] technical challenges in laparoscopic Hill repair for GERD were addressed through real-time intraoperative HREM monitoring of LES pressure, aiming to maintain it within 25–35 mmHg. This approach provided critical feedback on suture tightness, leading to satisfactory patient outcomes post-surgery after a 6-month follow-up.

Despite HREM’s promising applications in both preoperative and postoperative diagnostics and prognostics for GERD, ongoing research is essential to fully establish its clinical value.

## 6. Predicting PPI Efficacy in GERD Based on HREM

Recent research has revealed that individuals who do not respond to PPI treatment are more likely to have an intact EGJ barrier function and a higher level of esophageal body contractile vitality, and these patients also exhibit a lower degree of acid reflux burden and experience persistent symptoms, potentially implicating non-GERD mechanisms [65]. Y. Shi et al. [66] found that patients with PPI treatment failure frequently presented with type I EGJ morphology, enhanced EGJ augmentation, and a higher prevalence of esophageal motility disorders. These observations suggest that HREM findings can serve as predictive indicators for PPI treatment effectiveness and contribute to a deeper understanding of refractory GERD symptoms.

## 7. Predicting Acid Exposure and Assessing GERD Severity Based on HREM

GERD, characterized by the retrograde flow of gastric acid into the esophagus, primarily varies in severity based on the degree of esophageal acid exposure. This exposure is a consequence of compromised anti-reflux barrier function and reduced esophageal clearance capacity, leading to reflux disease development.

Certain manometric parameters, indicative of anti-reflux capabilities and esophageal clearance efficiency, can predict abnormal esophageal acid exposure. Rengarajan et al. [13] conducted a study in which they extracted AET from dynamic reflux monitoring of 482 patients. They also analyzed EGJ-CI and DCI extracted from HREM. Their findings established that HREM-based EGJ morphology, EGJ-CI, and DCI were independent predictors of abnormal AET. In addition, the reflux burden of IEM patients with a lower resting LES pressure and a higher percentage of swallowing impairment exhibited a more severe reflux burden [38].

GERD severity is classified into four grades (A to D) according to the Los Angeles (LA) Classification System. There is a noted correlation between higher LA grades and lower LES pressure, indicating the presence and severity of GERD [67,68]. Additionally, from NERD to severe esophagitis, the incidence of HH increases significantly, suggesting a direct relationship between GERD severity and the presence of HH [69].

## 8. Advancement in HREM-Based Provocative Testing: The Supine Position Straight Leg Raise (SLR) Test

The SLR test, a novel HREM-based provocative test, involves the patient elevating one leg to at least 45° for a minimum of 5 s. This test is considered effective in increasing intra-abdominal pressure (IAP) if a 50% elevation in IAP is observed during the maneuver. The SLR test is instrumental in assessing the EGJ barrier function by quantifying changes in intra-esophageal pressure (IEP) as the IAP rises. In most individuals without pathological acid reflux, the LES and CD increase pressure during the SLR test, counteracting gastric content regurgitation into the esophagus. However, this protective mechanism is impaired in GERD patients, leading to an elevated IAP that is transmitted to the esophageal body, thus exacerbating the reflux burden.

In a study by Siboni et al., GERD patients exhibited higher peak IEP values during the SLR test compared to non-GERD individuals (29.7 vs. 13.9 mmHg). Furthermore, regardless of HH presence, an 11 mm Hg increase in peak IEP during the SLR test over baseline was identified as the optimal value for predicting an AET greater than 6% (sensitivity 79%; specificity 85%) [70]. In cases of ineffective esophageal motility, a significant association was found between transient esophageal hiatus separation (separation between LES and diaphragm ≥ 1 cm) during the SLR test and higher AET, DeMeester scores, total acid reflux events, and prolonged gastric acid reflux episodes) [71].

The SLR test has been validated in multiple studies for its efficacy in enhancing GERD diagnostic accuracy [70,72], predicting esophageal contraction reserve [73], and assessing the extent of the reflux burden [70,71,74,75]. Its diverse applications make it a promising tool in the diagnostic and therapeutic landscape of GERD. However, it is not yet a routine part of clinical practice. As per the Chicago Classification 4.0 guidelines, this provocative test may be conducted if no severe esophageal motility disorder is detected during standard manometry. Nonetheless, there is still a lack of widespread consensus on its routine clinical implementation.

## 9. Advancements in GERD Assessment Using HREM-Based Technologies

### 9.1. Combined Impedance Monitoring–High-Resolution Impedance Manometry (HRIM)

HRIM combines an HREM catheter with an impedance sensor, allowing the simultaneous assessment of food bolus transport and esophageal pressure dynamics. It is a useful tool to diagnose masqueraders of reflux, such as rumination syndrome, primary motility disorders, and esophagogastric junction [8]. By evaluating bolus emptying, reflux characteristics, and esophageal clearance, HRIM offers a multifaceted understanding of esophageal motility. When employing a baseline impedance (BI) threshold of 1582 Ω in HRIM, its diagnostic efficacy for GERD includes a sensitivity of 86.2%, specificity of 88.5%, positive predictive value of 89.3%, and a negative predictive value of 85.2% [76]. A study revealed a negative correlation between systolic segment impedance (CSI) measurements in HRIM and AET, suggesting that CSI could effectively differentiate between pathological and physiological AET, thereby emerging as a reliable, efficient, and minimally invasive marker for GERD diagnosis [77].

Postprandial HRIM (PP-HRIM) is conducted following a standardized test meal (STM) or symptom-eliciting meal, lasting at least 10 min [14]. Normal PP-HRIM is characterized by symptom absence during postprandial monitoring and no abnormal power or functionality indications. Within the initial 10 min post-meal, it records no more than four TLESR instances accompanied by belching, and no events of volume reflux, rumination, or gastric belching. Yadlapati et al. [78] utilized PP-HRIM for assessing 94 PPI-resistant GERD patients, demonstrating its capability to distinguish reflux types, rumination, and belching and elucidate the causes of PPI unresponsiveness, including acid, weak acid, and non-acid reflux. Thus, PP-HRIM serves as a valuable clinical tool for assessing pathophysiology in GERD patients unresponsive to PPI therapy, guiding appropriate treatment approaches.

### 9.2. 3D High-Resolution Pressure Measurement with HREM

Three-dimensional high-resolution manometry (3D-HREM) employs a multi-sensor catheter and software for 3D reconstruction and analysis of esophageal function. This approach offers detailed anatomical visualization and dynamic characterizations, facilitating simultaneous measurement and anatomical positioning.

The 3D-HREM’s application for evaluating the EGJ functional barrier is noteworthy. It provides real-time recordings of EGJ pressure morphology, enabling the analysis of specific EGJ components that are crucial for its reflux barrier function. This positions 3D-HREM as an innovative tool in GERD pathophysiology research [79]. Several studies highlight the advantages of assessing the UES using 3D-HREM over traditional HREM, contributing significantly to our understanding of UES physiology [80,81].

## 10. Esophageal Motility Assessment Utilizing the Endolumenal Functional Lumen Imaging Probe (EndoFLIP)

EndoFLIP represents a cutting-edge endoscopic technology, particularly for patients with GERD. It involves the placement of a balloon device at the EGJ to quantitatively measure internal pressure and cross-sectional area in real time. These data are then transformed into cylindrical shapes representing various internal diameters, effectively visualizing EGJ pressure and compliance. The key parameters measured include cross-sectional area (CSA, in mm^2^), diameter (in mm), distensibility index (DI, in mm^2^/mm Hg), intra-bag pressure (in mmHg), and esophageal contractions elicited by balloon distension, such as repetitive antegrade contractions (RAC), repetitive retrograde contractions (RRC), diminished disordered contractile response (DDCR), and absent contractility. EndoFLIP’s primary function is to assess the EGJ’s scalability and has been extensively used in the diagnosis of esophageal functional disorders. Normally, the EGJ-DI should be ≥2.8 mm^2^/mm Hg, and the esophageal body should exhibit RAC [82].

Hoppo et al. [83] highlighted EndoFLIP’s capability of measuring and visualizing tissue distensibility changes at the EGJ, suggesting its potential as a single testing modality for diagnosing GERD and evaluating post-surgical outcomes. Although studies have indicated EndoFLIP’s utility in objectively diagnosing GERD and classifying its subtypes, [84] and GERD patients tend to exhibit higher EGJ distensibility than healthy individuals, its effectiveness in GERD diagnosis remains a subject of debate. Nonetheless, EndoFLIP’s ability to measure EGJ distensibility is seen as a promising tool in GERD diagnosis.

In the context of typical achalasia symptoms without standard HREM features (normal IRP) [85,86], EndoFLIP has been effective in detecting abnormal EGJ dilation, complementing HREM in the identification of EGJ outflow-tract obstructive diseases [7,87]. Moreover, during surgical procedures, EndoFLIP provides real-time objective feedback. As Min P. Kim et al. suggested, it can be used during fundoplication to tailor crus closure and fundoplication, enabling a personalized surgical approach [88]. Additionally, it assists in customizing gastric fundoplication or endoscopic anti-reflux surgery and in evaluating surgical outcomes [7,83,89,90,91]. A study involving 175 patients found that reducing intraoperative EGJ-DI to less than 2 mm^2^/mmHg was associated with postoperative dysphagia and bloating, whereas maintaining EGJ-DI between 2–3.5 mm^2^/mmHg led to more favorable outcomes [92]. EndoFLIP has also been shown to predict surgical response to peroral endoscopic myotomy (POEM) and postoperative reflux in achalasia patients [93]. Furthermore, Van Druff et al. compared preoperative EndoFLIP results with HREM in 120 anti-reflux surgery patients, proposing EndoFLIP as an alternative to HREM, especially when HREM is inaccessible or poorly tolerated [94]. EndoFLIP thus plays a crucial role in evaluating anti-reflux barrier function, guiding surgical decisions, and improving outcomes (Table 3).

## 11. Conclusions

Recent advancements in technology have significantly elevated our understanding, diagnostic precision, and treatment efficacy for GERD, as well as our ability to distinguish it from other disorders. The integration of innovative equipment, technologies, and methodologies has deepened our insights into the mechanisms of reflux and esophageal motility. However, the analysis and interpretation of esophageal manometry data often require manual intervention, and the absence of standardized thresholds and algorithms complicates the calculation processes. While HREM has established data standards, the application parameters for EndoFLIP are less uniform and necessitate further consolidation.

Future research should focus on conducting well-designed studies in specialized centers with large sample sizes. This will further enhance the generalizability, applicability, and stringent diagnostic criteria of esophageal motility testing. The overarching goal is to improve the diagnosis, treatment, and overall management of GERD, aligning with the evolving landscape of medical technology and patient-centered care.

## Figures and Tables

**Table 1:** Utility of HREM in GERD.

Application	Representation	Main Parameters Involved
Understanding pathophysiologies	Impairment of the anti-reflux barrier	LES, CD, EGJ-CI
	TLESR	LES, CD
	Motor disorders of the Esophageal body	DCI
Auxiliary diagnosis	the decrease of EGJ pressure,	LESP
	HH	LES, CD
	IEM/absent contractility	DCI
Differential diagnosis of esophageal motility disorders	EGJOO	IRP, DCI
	AC	IRP
	esophageal hypermotility	DCI
	DES	DCI, DL
Guide anti-reflux surgical	-	DCI, MRS, IRP, LES, CD, LESP
Predicting the efficacy of PPI	-	DCI, LES, CD
Predicting acid exposure and severity	-	EGJ-CI, DCI, LES
Application of new equipment	HRIM	BI, CSI
	3D-HREM	-

LES Lower esophageal sphincter, CD crural diaphragm, EGJ-CI esophagogastric junction-contractile integral, DCI distal contraction integral, TLESR transient lower esophageal sphincter relaxation, IRP Integrated relaxation pressure, DL distal latency, DES distal esophageal spasm, MRS multiple rapid swallows, LESP lower esophageal sphincter pressure, PPI proton pump inhibitors, EGJ esophagogastric junction, BI baseline impedance, CSI contractile segment impedance, HRIM high-resolution impedance manometry, 3D-HREM Three-dimensional high-resolution manometry, GERD gastroesophageal reflux disease.

**Table 2:** Comparative analysis of EGJ-CI thresholds and methodologies in various studies.

Study	Subject Groups	Threshold	Algorithm	Conclusion
Humayra Dervin et al. [31], 2023	BE (*n* = 25) NERD (*n* = 25) AET4–6% (*n* = 25) FH (*n* = 25)	21.2 mmHg·cm	Recommend threshold 20 mmHg above gastric pressure	The distinction between Barrett’s esophagus/NERD and FH had a sensitivity of 72% and a specificity of 72%.
Jasper D et al. [24], 2017	HC (*n* = 65) GERD (*n* = 452)	47 mmHg·cm	threshold 2 mmHg above gastric pressure	The sensitivity of GERD diagnosis was 54%, and the positive predictive value was 46%.
Rengarajan A et al. [13], 2020	GERD (*n* = 482)	39.3 mmHg·cm	threshold 0 mmHg above gastric pressure	The EGJ-CIs were independent predictors of AET abnormalities.
S.Tolone et al. [10], 2015	GERD (*n* = 91) FH (*n* = 39)	13 mmHg·cm	threshold 2 mmHg above gastric pressure	The EGJ-CI is associated with AET, reflux episodes, and mucosal injuries. And, a cut-off value of 5 has the highest sensitivity (89%) and specificity (63%) in distinguishing GERD from FH.
Gor, P et al. [28], 2016	normal controls (*n* = 21), GERD (*n* = 188)	39.3 mmHg·cm	gastric baseline (rather than correction to a value above the gastric baseline) during a period of quiet rest	EGJ-CI is a novel HREM metric that has potential to complement or replace currently used basal LES and EGJ parameters.
Nicodème F et al. [32], 2014	normal controls (*n* = 75) PPI-NRs (*n* = 88)	39 mmHg·cm	threshold 2 mmHg above gastric pressure	The EGJ-CI may help distinguish between PPI-NRs patients with functional heartburn and patients with refractory GERD.
Benjamin D Rogers et al. [30], 2021	Health volunteers (*n* = 484)	-	threshold 20 mmHg above gastric pressure	The 5th percentile EGJ-CI value was 6.9 to 12.1 mmHg·cm.
D. WANG et al. [33], 2016	Twenty-one achalasia patients, 68 GERD patients, and 21 healthy controls	-	gastric baseline (rather than correction to a value above the gastric baseline) during a period of quiet rest	The EGJ-CI has clinical utility in assessing EGJ barrier function at baseline and after surgical intervention to the EGJ.

EGJ-CI esophagogastric junction contractile integral, BE Barrett’s esophagus, FH functional heartburn, NERD non-erosive reflux disease, AET acid exposure time, HC healthy controls, PPI-NRs proton pump inhibitor non-responders.

**Table 3:** Evaluating the utility of EndoFLIP in GERD management.

References	Population	Volumes Distension	Process	Findings
**Diagnosis**				
Tucker et al. [95], 2013	Twenty-one HV and 18 patients with typical GERD symptoms	20–30 mL	enteral anesthesia	EndoFLIP technology cannot be used to diagnose GERD.
Lee et al. [84], 2021	ERD (*n* = 204), NERD (*n* = 310), and 277 normal subjects	40 mL	not very clear	The measurement of EGJ distensibility was helpful in the diagnosis of GERD. The EGJ distensibility of GERD patients was higher than that of normal subjects, regardless of the presence of reflux esophagitis.
Carlson et al. [96], 2018	25 patients	10–70 mL	conscious sedation	No correlation was found between EGJ-DI and reflux parameters including AET, number of reflux episodes, and longest reflux episodes.
Smeets et al. [97], 2015	GERD (*n* = 42) and 25 patients receiving TIF treatment were followed up for 6 months	20–30 mL	- Induction general anesthesia and conscious sedation	EndoFLIP technique has no additional value in preoperative diagnosis.
Kwiatek et al. [98], 2010	GERD (*n* = 20) and controls (*n* = 20)	10–40 mL	- Esophagogastroduodenoscopy, conscious sedation	GERD patients exhibited two- to threefold increased EGJ distensibility compared with controls, particularly at 20 to 30 mL distention volumes.
**Differential Diagnosis**				
Carlson et al. [99], 2022	asymptomatic volunteers (*n* = 35), primary esophageal motility evaluation patients (*n* = 687)	40–70 mL	Esophagogastroduodenoscopy, conscious sedation	Normal EGJ opening: EGJ-DI > 2–3 mm^2^/mm Hg and EGJ diameter > 12–16 mm, EGJ-DI < 2 mm^2^/mm Hg and EGJ diameter < 12 mm suggest EGJ outflow obstruction)
Ponds et al. [85], 2017	13 patients of achalasia	20–50 mL	Conscious without sedation	EGJ distensibility measured can diagnose achalasia despite normal IRP (<15 mmHg).
**Anti-reflux surgical**				
Julia R. Amundson et al. [100], 2023	TFHB (*n* = 147), TFFB (*n* = 69), TFNB (*n* = 78), NFHB (*n* = 20), NFFB (*n* = 19)	30–40 mL (8 cm) 60 mL (16 cm)	general anesthesia	The CCDI > 3.5 mm^2^/mmHg (40 mL fill) should be sought in abnormal motility patients, regardless of wrap or bougie, to avoid postoperative dysphagia. TFFB abnormal motility patients with FDI > 3.6 mm^2^/mmHg (40 mL fill) also developed zero postoperative dysphagia. FDI > 6.2 mm^2^/mmHg (40 mL fill) was seen in all postoperative hernia recurrences.
Wu, H et al. [101], 2022	GERD (*n* = 111) (26% Nissen, 74% Toupet)	30–40 mL	Intra-operative, General anesthesia	A LON of 2.5–4.5 cm and DI of 2.5–3.6 mm^2^/mmHg after fundoplication led to better postoperative quality of life.
Min P. Kim et al. [88], 2018	Forty patients underwent minimally invasive hiatal hernia repair with fundoplication	30 mL	Intra-operative, General anesthesia	EndoFLIP can be used to help tailor how tight to close the crus and how tight to create the fundoplication. (decided to aim for a DI > 0.5 mm^2^/mm Hg, as measured with 30 mL in the balloon.)
Wu, H et al. [102], 2022	Two hundred fifty patients (171 Toupet, 79 Nissen)	30–40 mL	Intra-operative, General anesthesia	The ideal distensibility index range of Toupet patients with the 30 and 40 mL balloon fills was 2.6 to 3.7 mm^2^/mmHg. For Nissen patients, the 30 and 40 mL ideal threshold was a distensibility index of ≥2.2 mm^2^/mmHg.
Su, B et al. [92], 2019	175 patients underwent laparoscopic fundoplication	20–40 mL	Intra-operative, General anesthesia	EndoFLIP measurements correlate well with patient outcomes, with a final DI between 2 and 3.5 mm^2^/mmHg potentially being ideal. And EndoFLIP measurements correlate well with patient outcomes.
DeHaan et al. [103], 2017	75 patients underwent fundoplications	30–40 mL	conscious sedation	EGJ distensibility can be determined in real-time intraoperatively and that fundoplication results in a decreased distensibility of the EGJ in patients with GERD.
Monika et al. [104], 2010	Ten controls and ten Nissen FP patients were studied	30–60 mL	conscious sedation	After FP, the stretchability of EGJ decreased and the shrinkage length increased.
Smeets et al. [97], 2015	42 GERD patients and 25 patients receiving TIF treatment were followed up for 6 months	20–30 mL	induction of general anesthesia and conscious sedation	EndoFLIP technology has no added value in the postoperative evaluation of endovascular anti-reflux therapy.
Turner et al. [105], 2020 entry 4	Forty-three GERD patients	30 mL	general anesthesia	The EndoFLIP probe is a useful tool that can provide feedback during gastric fundus folding surgery and allows surgeons to customize the geometry of the package to optimize symptom outcomes.

HV healthy volunteers, CSA cross-sectional area, BMI body mass index, ERD erosive reflux disease, NERD non-erosive reflux disease, AET acid exposure time, TFHB Toupet with hard bougie, TFFB Toupet with EndoFLIP bougie, TFNB Toupet without bougie, NFHB Nissen with hard bougie, NFFB Nissen with EndoFLIP bougie, CCDI distensibility index at crural closure, FDI distensibility index at post-fundoplication, LON length of narrowing, DI distensibility index, Dmin minimum diameter, FD fundoplication, TIF transoral incisionless fundoplication.

## Data Availability

Not applicable.

## Abbreviations

GERD, gastroesophageal reflux disease; RE, reflux esophagitis; NERD, non-erosive reflux disease; PPI, proton pump inhibitor; LES, lower esophageal sphincter; HREM, high-resolution esophageal manometry; rGERD, refractory GERD; UES, upper esophageal sphincter; CD, crural diaphragm; DCI, distal contraction integral; IRP, integrated relaxation pressure; DL, distal latency; EGI-CI; esophagogastric junction-contractile integral; TLESR, transient lower esophageal sphincter relaxation; DES, distal esophageal spasm; MRS, multiple rapid swallows; LESP, lower esophageal sphincter pressure; EGJ, esophagogastric junction; BI, baseline impedance; CSI, contractile segment impedance; HRIM, high-resolution impedance manometry; 3D-HREM, three-dimensional high-resolution manometry; EndoFLIP, Endoscopic functional lumen imaging probe; HH, Hiatal hernia; FH, functional heartburn; AET, acid exposure time; IEM, ineffective esophageal motility; AC, achalasia of cardia; EGJOO, esophagogastric junction outlet obstruction; RDC, rapid drink challenge; STM, solid test meal; DI, distensibility index; CSA, cross-sectional area; RAC, repetitive antegrade contractions; RRC, repetitive retrograde contractions; DDCR, diminished disordered contractile response; POEM, peroral endoscopic myotomy; DHPZ, identifying dual high-pressure zones; SLR, straight leg raise; IAP, intra-abdominal pressure; IEP, intra-esophageal pressure; PP-HRIM, postprandial HRIM
